# Current and future developments of synthetic computed tomography generation for radiotherapy

**DOI:** 10.1016/j.phro.2023.100521

**Published:** 2023-11-15

**Authors:** Wouter van Elmpt, Vicki Trier Taasti, Kathrine Røe Redalen

**Affiliations:** a Department of Radiation Oncology (MAASTRO), GROW School for Oncology and Reproduction, Maastricht University Medical Center+, Maastricht, The Netherlands; b Department of Physics, Norwegian University of Science and Technology, Norway

The literature on generation of synthetic computed tomography (CT) based on magnetic resonance imaging (MRI) or cone-beam CT (CBCT) has been rapidly increasing over the last years [1], [2], [3], [4]. Recently, also commercial solutions for synthetic CT generation have been introduced, advancing adaptive radiotherapy workflows [5], [6], [7], [8], [9], [10], [11]. The interest in MRI-only workflows with synthetic CTs replacing the planning CT is also growing [12].

With the clinical introduction of synthetic CTs, there has been a shift in the literature. While the first papers in this field generally focused on image-based assessments only and mainly reported on CT number accuracy [13], [14], recent papers are focusing on evaluating the entire chain of the radiotherapy process [15], [16]. Linked to this, the time it takes for these algorithms to perform the corrections also becomes an important part of the evaluation. Besides image quality and geometrical correctness, a dose/volume-based evaluation by (re)calculation of treatment plans on synthetic CT scans is currently seen as one of the standard methods to quantify the accuracy of a synthetic CT generation [17], [18].

With the aim of establishing a patient-specific method for quality assurance, Dal Bello et al. [15] in this ESTRO 2023 paper highlight collection compared four commonly used strategies to create a synthetic CT from the MRI; ranging from a very simplistic assumption of overriding the entire patient inside the body contour with water to advanced correction methods based on deformable image registration (DIR) or deep-learning based neural networks. Creating a separate synthetic CT based on an independent neural network was found to provide an efficient and accurate validation method; however, for patients with metal implants the MR-based synthetic CTs were less reliable.

In another ESTRO 2023 highlight paper, Texier et al. stressed the need for using multi-center data also in the training step of the deep-learning models [16]. They performed an investigation using data from four different centers to create a synthetic CT model for MRI. Here the generalizability was poor if the model was applied on scans from a different site that was not included in the model. This is important to realize when robust deep-learning synthetic CT models are intended to be used across multiple MRI scanners, protocols, or institutes.

For CBCT based adaptive therapy, the synthetic CT has also shown to be a powerful tool in the evaluation of treatment quality. Typically, the image quality of CBCT is insufficient for accurate dose calculation. Synthetic CT approaches have shown to be able to allow for dosimetric assessment of treatment planning for example in the lung [19], head-and-neck [20], or simulating prostate motion [21]. Also, novel approaches using the diagnostic CT in combination with the CBCT in a palliative setting has been explored [22].

However, to be able to safely use synthetic CTs in an (online) adaptive workflow, it is important to ensure that the synthetic CTs represent the geometry of the patient correctly. With frequently used DIR-based algorithms, this geometrical accuracy could be investigated and steered by the regularization of the deformation vector field. Currently, many CBCT-based synthetic CT generation methods are based on deep-learning which are trained on both planning CT and CBCT scans which may not represent the same anatomy of the patient. It should therefore be evaluated if the synthetic CT method only improves the image quality of the CBCT without introducing anatomical differences. In a study by de Hond et al., it was found that among three deep-learning synthetic CT methods for CBCT conversion the method giving the best image quality performance, based on mean absolute error of CT numbers, was different from the method giving the best anatomical performance, based on organ-at-risk volumes and average surface distance [23].

Furthermore, for moving tumors the treatment plan is often based on a 4D-CT, whereas in the treatment room typically only 2D or 3D imaging is available. Deep-learning methods have been proposed to generate 3D-sCTs based on 2D radiographs [24], or even to generate 4D-sCTs based on 3D-CBCTs [25]. The integration in a clinical routine workflow is however not yet fully investigated.

For proton therapy, MRI-only treatment planning is also advantageous in order to bypass the MR-CT co-registration and to benefit from the improved soft tissue contrast. However, MRI alone cannot provide stopping-power ratio (SPR) information. Recognizing that dual-energy CT can estimate SPR better than single-energy CT, Liu et al. [26] demonstrated a new method using deep-learning to create synthetic dual-energy CT from MRI to calculate SPR. Hence, it is likely that synthetic CTs will find their way into both photon- and proton therapy in the years to come, closely linked with development of novel artificial intelligence algorithms.

The commercial synthetic CT solutions often originate from developments of in-house scripts, finally being translated to clinical innovation. It is yet another example of how physics-driven research result in new technological solutions for the benefit of further personalization and improvements of cancer treatment. It can be anticipated that the current hurdles with standardization and validation will be solved. At the moment, the preferred validation is ideally based on time-consuming collection of data from multiple institutions to make the models robust. In the future, either federated learning solutions, or other novel ways could be envisioned through an integrated approach of creating unlimited privacy-safe synthetic images for the purpose of validation.

## Declaration of Competing Interest

The authors declare that they have no known competing financial interests or personal relationships that could have appeared to influence the work reported in this paper.
